# Human Milk Composition: An Atlas for Child Health Recommendations

**DOI:** 10.1016/j.advnut.2023.100151

**Published:** 2023-11-17

**Authors:** Ashley J. Vargas

**Affiliations:** Division of Extramural Research and Training, National Institute of Environmental Health Sciences, Durham, NC, United States

There is strong evidence that breastfeeding (chestfeeding) and/or human milk consumption improve child and family health outcomes, and many hypotheses as to why this relationship exists, including improved family bonding, protecting maternal health, and the transfer of beneficial human milk components to the infant [1]. Of all these potential hypotheses, perhaps the most straightforward to test is the relationship between human milk composition and health outcomes, including normal growth and development. However, human milk contains thousands of components, and it varies greatly between and even within lactating individuals. Additionally, assessing components of human milk is technically challenging [2]. Thus, although human milk is recommended as an infant’s first food [3], we know very little about what, and how much, is in human milk.

Human milk composition plays a key role in setting public health and medical recommendations. All infants deserve access to safe and nutritious early food, and so one major role for knowledge on what components of human milk benefit health is to inform recommendations for infants who do not have adequate access to human milk [4, 5]. For children over age 6 mo, it is recommended that they meet their nutrient requirements from both human milk consumption and the gradual introduction of foods and beverages. Therefore, in order to determine which foods and beverages should be recommended to complement human milk intake with a goal of meeting nutrient needs, there is a need to understand what and how much of each nutrient human milk provides. Lastly, some human milk composition is linked to exposures (including diet, body composition, and the environment) of the lactating individual [6], making the surveillance and understanding of human milk composition overtime an important public health need [4].

Despite its importance, there has historically been a lack of data on human milk due to the challenges mentioned above and an insufficient level of resources to support this kind of research [4, 7]. This has made updating crucial nutrition guidelines, like the Dietary Reference Intakes (DRIs) and Dietary Guidelines for children, impossible in some situations. For example, due to a lack of data on human milk, the 2020-2025 Dietary Guidelines for Americans does not include dietary patterns for children ≤ 1 y of age, and the DRIs for children ≤ 1 y are mostly Adequate Intakes, which are the lowest quality recommendations possible for a DRI. To this end, there have been several efforts to survey the available data on nutrients in human milk in the published literature to glean what information is available for guideline development and where the research gaps are [4, 5].

Moving beyond just surveying the data available, Brockway et al. (macronutrients) [8], Reyes et al. (micronutrients) [9], and Brockway et al. (other bioactives) [10] have provided a comprehensive evaluation and summation of the available published data on human milk composition between 1980 and 2022. Their findings highlight a paucity of high-quality studies that analyze human milk nutrient and other bioactive composition, and a body of literature that is rife with too much heterogeneity. These challenges limit the ability to derive a summary set of values that could be used for guidelines development. My hope is that researchers and funders will use this exhaustive set of manuscripts to justify the prioritization of resources toward comprehensive evaluations of human milk composition, in a representative population and across time to improve our ability to provide recommendations to the youngest members of our population.

## Conflicts of interest

The author is an US federal government employee and has no conflicts of interest to declare.

## Funding

None.
